# Exploring the Feasibility of an Online Diabetes Wellness Programme among Periodontitis Patients with Type II Diabetes Mellitus during the COVID-19 Pandemic

**DOI:** 10.3390/healthcare10112129

**Published:** 2022-10-26

**Authors:** Nur Fadzlin Syahira Rusly, Nor Aini Jamil, Tuti Ningseh Mohd-Dom, Haslina Rani, Shahida Mohd-Said, Nur Adila Mohd-Norwir, Afendi Hamat

**Affiliations:** 1 Centre for Community Health Studies (ReaCH), Faculty of Health Sciences, Universiti Kebangsaan Malaysia, Kuala Lumpur 50300, Malaysia; 2 Department of Family Oral Health, Faculty of Dentistry, Universiti Kebangsaan Malaysia, Kuala Lumpur 50300, Malaysia; 3 Centre for Restorative Dentistry, Faculty of Dentistry, Universiti Kebangsaan Malaysia, Kuala Lumpur 50300, Malaysia; 4 Centre of Language and Linguistics, Faculty of Social Sciences and Humanities, Universiti Kebangsaan Malaysia, Bangi 43600, Malaysia

**Keywords:** diabetes, wellness, nutrition, online education, oral health

## Abstract

There were massive interruptions, including patient visits for dietary advice and dental services, during the COVID-19 outbreak. This study assessed the feasibility of an online diabetes wellness programme among periodontitis patients with type II diabetes mellitus. Patients were grouped into the intervention group (IG) and control group (CG). At baseline and week 12, all patients answered online questionnaires on diabetes-related knowledge, physical activity, and oral impacts on daily performances (OIDP). Body weight and waist circumference were self-measured with guided instructions. Diet recalls were used to estimate dietary and added sugar intake. All patients received a weekly educational video, but the IG attended three e-consultation sessions with a dietitian (weeks 1, 3, and 8) and a dentist (week 8) via video call. A semi-structured interview was conducted to collate qualitative feedback among the IG participants at the end of the programme. A total of 24 periodontitis patients (14 IG and 10 CG) participated in this study. Among the IG patients, significant improvements in diabetes knowledge, body weight, BMI, calories, carbohydrates, fat, and added sugar intakes were observed at week 12. The CG patients only had a significant reduction in carbohydrate intake post intervention. No changes were reported in physical activity level and OIDP for both groups. Feedback received from the IG participants included convenience, practical, preferred approach during the pandemic, increased awareness and knowledge, and favourable lifestyle changes. This study demonstrates that an online diabetes wellness programme with healthcare professionals is feasible and can facilitate knowledge and lifestyle improvements that can be adapted during the crisis situation.

## 1. Introduction

Type II diabetes mellitus (T2DM) is a metabolic disease that can cause multiple organ damages and complications, while periodontitis is one of the silent complications of T2DM [1,2]. Previous studies established a bidirectional relationship between T2DM and periodontitis [1,3,4]. While there are distinct medical conditions between these two diseases, they may worsen each other through biochemical mechanisms at the cellular and molecular levels. Periodontitis exacerbates and dysregulates the inflammatory response, leading to poor blood sugar control and increased insulin requirements. Interleukin-6 (IL-6), tumour necrotizing factor-α (TNF-α), C-reactive protein (CRP), and oxygen radicals are the mediators between periodontal inflammation and glucose homeostasis [3,4]. In addition, periodontitis is more common and severe in patients with diabetes than in people without diabetes. Management of either disease may assist treatment of the other. In addition, diabetes screening is recommended for periodontitis patients, and newly diagnosed diabetes patients are advised to undergo a periodontal examination [4,5]. The national surveillance in Malaysia highlighted the growing prevalence of T2DM and periodontitis among the younger generation [6,7]. This condition calls for meticulous intervention to combat both diseases. However, there has been no established standard of care in the country to address both diseases’ management since highlighted in 2012 [8].

Healthcare providers have increasingly concerned that the restrictions posed during the COVID-19 pandemic may result in poorer health outcomes in the community due to massive disruptions in the healthcare systems, including patient appointments for dietary consultations and dental treatments. Individuals with T2DM may also experience a change in their exercise routine and dietary intake due to the lockdown [9]. In addition, individuals with co-morbidities, such as diabetes, are at a higher risk of incident mortality and severe infection from COVID-19 than those who do not have diabetes [10]. Moreover, people who had COVID-19 are more likely to develop diabetes, and the lack of regular dental visits during the pandemic increased the prevalence and severity of periodontitis [11,12]. Thus, patients with diabetes and periodontitis must prioritise self-health care to mitigate the impact of the pandemic.

Online education programmes could provide ongoing support for lifestyle changes during the pandemic. Evidence supporting the efficacy of a telemedicine-based intervention in promoting diet changes during non-pandemic situations was reported previously [13]. Therefore, this study aimed to assess the feasibility of an online diabetes wellness programme on lifestyle changes during the COVID-19 pandemic among T2DM patients with periodontitis.

## 2. Materials and Methods

### 2.1. Study Design

**Figure 1:**
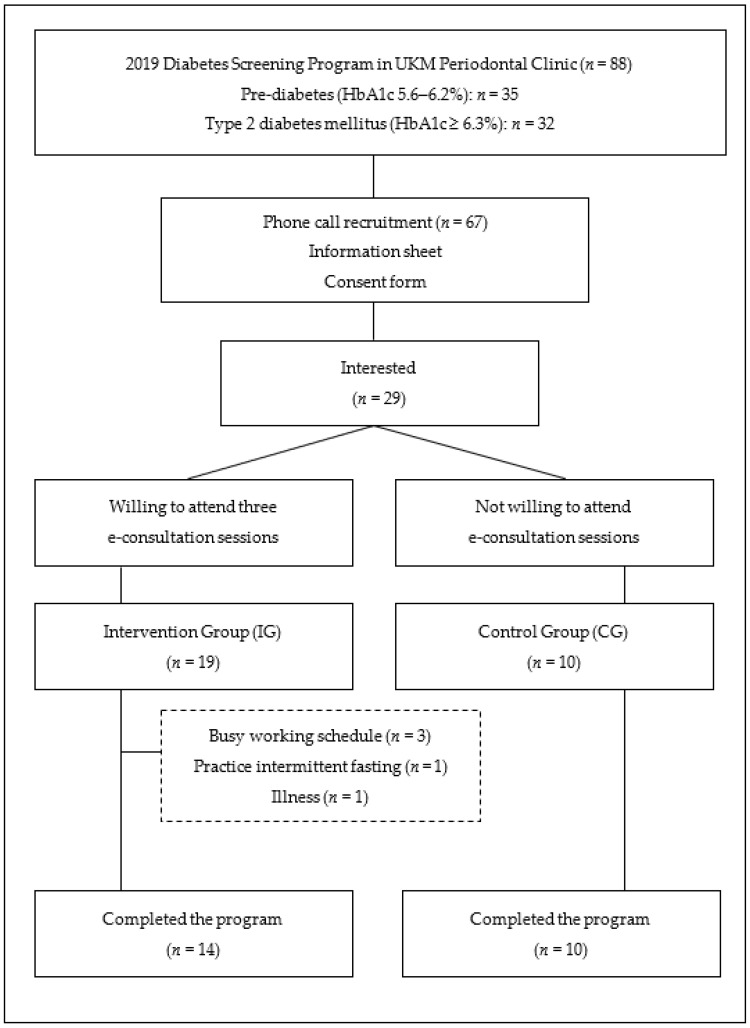
Participants’ enrolment.

This experimental study used a purposive sampling technique among periodontitis patients receiving dental treatment in a periodontal clinic in Kuala Lumpur, Malaysia. In 2019, 88 periodontitis patients were screened for HbA1c levels, and 67 patients were found to have pre-diabetes or T2DM. In March 2020, the government of Malaysia implemented a movement control order in the country as the number of COVID-19 cases increased. Therefore, the researchers transformed the initial plan to conduct a diabetes wellness programme at the clinic into an online education programme. All periodontitis patients with pre-diabetes and T2DM (*n* = 67) were invited by phone to this online educational programme (Figure 1). Only 29 patients (43%) expressed their interest in participating, with 19 patients agreeing to attend the e-consultation sessions and assigned to the intervention group (IG). The remaining patients were assigned to the control group (CG). However, five IG patients dropped out of the study due to time constraints (*n* = 3), illness (*n* = 1), and decided to practice intermittent fasting (*n* = 1).

Eligibility criteria for the study included those aged 18 years and older, diagnosed with periodontitis and pre-diabetes/T2DM, good command of the Malay language, and adequate access to telecommunication devices such as smartphones, computers, or tablets with a good internet connection. Patients with other underlying diseases such as type I diabetes mellitus or chronic kidney disease, pregnancy, impairments in hearing, sight or physical movement, and currently practising vegetarian or any special diet were excluded. This study received ethics approval from the University’s Research Ethics Committee (reference code: UKM/PPI/111/8/JEP-2020-0613), and all participants gave written informed consent before the start of the study.

### 2.2. Online Diabetes Wellness Programme

The platform for this programme was designed with three main requirements: speed of development, low cost of development, and ease of maintenance. A custom-developed solution would offer a more polished product; yet, it would not meet the first two requirements set by the research team. The diabetes wellness programme was built using the WordPress publishing system, and functions such as food diary and appointment sub-systems were developed using open-source and commercial plug-ins for WordPress. The versatility of WordPress reduced development time drastically, and it took the researchers less than a month to complete both the system development and training for the users.

**Figure 2:**
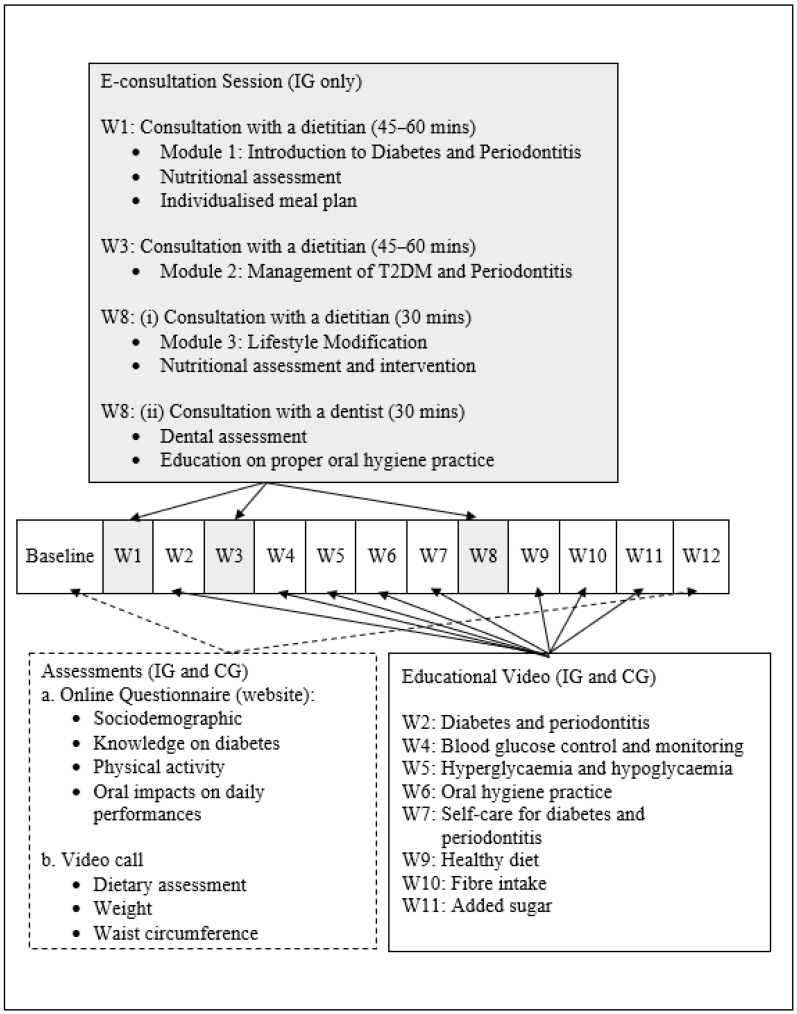
Diabetes Wellness Programme Module.

This programme was meticulously designed for a 12-week online education programme that included watching weekly educational videos for both groups (Figure 2). The IG patients received three e-consultation sessions via video chat with a dietitian (weeks 1, 3, and 8) and a dentist (week 8) as additional features. Healthcare professionals had previously validated the module used during the e-consultation sessions and the educational videos [14,15]. All conversations held during the e-consultations were recorded using Google Meet or Zoom with the participants’ permission.

### 2.3. Data Collection

Eligible participants received an individual ID and password to access the official website (https://jomsedia.my; accessed on 20 October 2022). Then, they answered the online questionnaires on socio-demographic profile, diabetes-related knowledge [16], physical activity [17], and a validated Malay language version of the Oral Impacts on Daily Performances questionnaire (OIDP) [18] at baseline and week 12 (Figure 2). Next, a video call session was conducted for the dietary assessment using multiple past 24-h diet recall (baseline and week 12) and a food frequency questionnaire for added sugar intake [19]. Data for calories and macronutrients were analysed based on the Malaysian food database [20] using the Nutritionist Pro^TM^ software, Axxya Systems LLC, Woodinville, WA, USA. Added sugar contents were estimated based on previously published work related to added sugar intake in Malaysia and the nutrition information panels provided by the participants [21,22,23].

Body weight was self-measured by the participants with guided instructions. Participants with a home weighing scale were encouraged to weigh themselves during the interview session, and those without a weighing scale were given pictorial instructions to weigh themselves at work or other locations and provided the information to the researcher within the baseline week and week 12. Height was taken from previous screening data and validated by participants during the baseline interview session. A measuring tape was provided via postage after participants agreed to participate in the programme. Each participant was asked to invite a family member or friend to measure waist circumference during the interview session, guided by the researcher. The measurement was performed twice, and the average of the recorded measurement was used. A third measurement was taken in the event of a difference >0.5 cm between the two measurements, and the average of the two closest measurements was used.

A semi-structured interview was conducted in week 12 with the IG patients to collate qualitative feedback on the online education programme. The interview covered two topics: (i) general opinion about the programme and (ii) barriers to adopting the recommended lifestyle practices. The researcher took field notes by writing on the scripts prepared during the interview session. The vocal tone, facial expressions, and any special occasions during the interview sessions were all recorded, as they could influence the findings. The responses were transcribed ad verbatim, and the transcripts were checked and compared with the original video files to identify any missing information, followed by thematic analysis using the NVivo 12 software programme. The themes were defined and refined, and the quotes were translated from Malay to English without changing their meaning.

### 2.4. Statistical Analysis

Statistical analysis was performed with the IBM SPSS software (version 25.0. IBM Co., Armonk, NY, USA). Data were checked for normality using the Shapiro–Wilk test, histogram, and scatterplot. Variables were described using mean and standard deviation or median and interquartile range as appropriate. Categorical variables were reported with number and percentage. An independent *t*-test was used to compare between groups. The paired *t*-test and Wilcoxon signed-ranked test (for not-normally distributed data) were used to determine the changes in diabetes knowledge, OIDP, and lifestyle practices between baseline and week 12.

## 3. Results

A total of 14 participants in the IG and 10 CG completed the online education programme. Patients in the CG were significantly younger (mean age = 41.1 years) and had higher education levels (all participants had tertiary education level) compared to the IG (mean age = 54.1 years, and only 28.6% had tertiary education level) (Table 1). Meanwhile, the IG participants had a longer periodontitis duration (median = 3.5 years) than the CG (2.0 years), but this was not statistically significant (*p* = 0.091). There were no other significant differences in socio-demographic profiles and medical history among the IG and CG patients at baseline.

### 3.1. Changes in Diabetes-Related Knowledge and Lifestyle Practices

Similarly, no significant differences were found between the IG and CG patients in the diabetes-related knowledge and lifestyle practices at baseline (Table 2). At the end of the programme, the IG patients had significant improvements in diabetes knowledge (M = 12%; *p* = 0.008), body weight (M = −1.5 kg; *p* = 0.039), BMI (M = −0.6 kg/m^2^; *p* = 0.029), calories intake (M = −605 kcal/day; *p* < 0.001), carbohydrate (M = −95 g/day; *p* < 0.001), fat (M = −24.9 g/day; *p* = 0.021), and added sugar intakes (M = −10.3 g/day; *p* = 0.016) (Table 2). The CG patients only had a significant reduction in carbohydrate intake (M = −64.8 g/day; *p* = 0.015) at post intervention. Both physical activity level and oral impacts on daily performance did not change from baseline to post intervention for both groups.

### 3.2. Qualitative Feedback on the Online Education Programme

Overall, the IG participants well-accepted the online diabetes wellness programme. The programme was perceived as convenient, as it saved their time and cost on commuting; made them feel more relaxed, comfortable, and safe; and was flexible (Table 3). The module was practical since it was easy to follow, involved two-way communication, and was informative. The participants also perceived that the online education programme was a preferred approach during the pandemic, increased awareness and knowledge, and brought positive changes in lifestyle practices.

The barriers to implementing the recommended lifestyle changes are summarised in Table 4. The internal factors were a lack of self-discipline and short programme duration, as they required a longer time to initiate and fully comply with the recommendations. Meanwhile, the external factor was a lack of support.

## 4. Discussion

The primary aim of this study was to assess the feasibility of an online diabetes wellness programme and initiatives to empower patients with diabetes and oral health self-care and explore their challenges and barriers to adopting a healthy lifestyle during the pandemic COVID-19. Feasibility was assessed using a few indicators on lifestyle modifications (quantitative approach) and patients’ views through interviews (qualitative). The programme was urgently transformed from a clinic visit to an online education programme when the pandemic first hit in Malaysia, and the government issued a movement control order. Most healthcare professionals had no experience with online consultation at that time. However, the online education programme requires participants to have adequate access to telecommunication devices such as smartphones, computers, or tablets with a good internet connection, which many do not have. Some patients had difficulty enrolling in this programme because they worked from home, and their children were involved in online learning using their phones/computers/tablets and had to share among siblings. These factors may explain the overall low participation rate and the preference for the CG (no online consultations) over the IG among younger and more educated patients, who were disproportionately affected by the lockdown order while working from home. Yet, despite the small sample size and demographic differences (older age, lower education level, and a tendency for longer periodontitis diagnosis in the IG), we discovered some lifestyle improvements and positive feedback from the IG, indicating a feasible approach to be adopted during the pandemic. Thus, this study serves as a pilot study and a proof of concept for the exploration of an online nutritional programme that has the potential to be used for patients during a crisis.

There was a significant improvement in diabetes knowledge among the IG patients at the end of the programme. Significant changes in dietary intake, particularly in energy, carbohydrate, fat, and added sugar intakes, were also observed among the IG. The CG showed a trend in knowledge improvements (*p* = 0.062) and a significant carbohydrate reduction at the end of the study. It is worth noting that both IG and CG received a weekly online educational video with topics ranging from understanding of the diseases to lifestyle and dietary recommendations. Only the IG received individual consultations with a dietitian and a dentist. This demonstrates that while online education can improve knowledge and lifestyle practices [24,25], individuals who receive personal consultation have a greater impact and sustainability than self-learning, as it offers more personalised advice, monitoring, and evaluation [26,27,28]. In addition, the patient-centred approach can increase patients’ motivation to make lifestyle changes [29].

The IG participants also had significant weight reduction at the end of the programme but no changes in their waistline. Contrary to our findings, a previous study found a significant weight loss and a reduction in waist circumference [30]. One possible explanation could be the inexperienced personnel measuring the waist despite receiving standard pictorial instructions [31].

However, neither group improved their physical activity level in the current study. According to a recent diabetes education programme in India, the leading cause of changes in physical activity levels observed in their study were rigorous behavioural support, continuous individual reinforcement, and weekly counselling strategies [32]. During the e-consultation session in our study, the IG participants only received individual suggestions on the type, duration, and frequency of physical activity. Hence, future programmes should incorporate a more enthusiastic approach to instil and reinforce physical activity. In addition, due to the movement control order enforcement during the study period, the participants might limit their physical exercise, as they were confined to their own homes during this time. It has been reported that physical activity decreased in all age groups worldwide during the COVID-19 pandemic regardless of gender [33].

Similarly, there was no significant change in the impact of oral health on daily performance for either group at the end of the programme. Despite a trend of improvement in the IG patients, it was not statistically significant (*p* = 0.085). This finding showed that clinical intervention is essential and should commence as soon as possible to improve oral health status among patients with periodontitis. Given the nationwide interruptions to dental services during the data collection, we could not conduct clinical assessments to ascertain the feasibility of this educational intervention on the periodontal status of these patients.

Tele-nutrition can be defined as remote, technology-supported visits (video or audio) to provide nutritional therapy to patients, including nutritional assessment, analysis, management plan, and monitoring and evaluation programmes [34]. The current global health situation urges the need to deliver nutrition education through various mediums, including the use of an e-consultation approach. However, regardless of the method used, healthcare professionals must ensure the targeted patients understand the key messages being delivered and take appropriate action to improve their lifestyles [35]. There were intrinsic barriers to lifestyle changes in the current study, including a lack of self-discipline and a short programme duration. Meanwhile, the external factor was a lack of social support. These barriers were also reported in a previous review on the barriers to making lifestyle changes among patients with T2DM, which include food habits, self-efficacy, emotional influence, motivation, social support, knowledge, socioeconomics, socio-cultural, environmental factors, and time management [36].

Overall, the participants received the current online diabetes wellness programme well; perceived it as a convenient, practical, preferred approach during the pandemic; increased their knowledge and awareness; and brought on lifestyle changes. A burden to travel and financial constraints associated with transportation was reported as a factor causing patients’ non-attendance at traditional clinic consultations [37]. A guideline has been proposed for physicians providing diabetes care through telemedicine during the pandemic to conduct the traditional face-to-face consultation session during the first encounter for physical examinations [35]. Such recommendations could also be employed in dietetics practice to improve the accuracy of physical measurements such as waist circumference.

This study has several limitations, including a small sample size and inclusion of only selected patients with periodontitis treated in a dental clinic in Kuala Lumpur, Malaysia. Thus, while the study’s findings could not be generalised to the general population and must be carefully interpreted due to the small sample size, it may serve as a proof of concept for exploring an online nutritional programme. This study also employed self-measured physical measurements, which could result in inaccuracy due to a lack of proper technique and a non-calibrated weighing scale. Finally, this study did not include any clinical periodontal or diabetes parameters due to the pandemic outbreak. Nonetheless, in view of these shortcomings, this pilot study could be a basis for assessing the feasibility of conducting online health education initiatives in the country.

## 5. Conclusions

The online diabetes wellness programme supported some lifestyle changes among periodontitis patients with pre-diabetes/T2DM during the pandemic COVID-19. Future research should assess the effectiveness and acceptance of this educational programme in a larger sample size. Our experience provides a model for integrated online education initiatives across health disciplines for improvements and wellness of patients with T2DM.

## Figures and Tables

**Table 1:** Socio-demographic profiles of the participants.

Characteristic	IG (*n* = 14)	CG (*n* = 10)	*p*-Value	Total (*n* = 24)
Age (years) ^a^	54.1 (13.0)	41.1 (11.5)	0.019	48.7 (13.8)
Sex ^b^			0.403	
Male	10 (71.4)	5 (50.0)		15 (62.5)
Female	4 (28.6)	5 (50.0)		9 (37.5)
Ethnic Group ^b^			0.580	
Malay	9 (64.3)	8 (80.0)		17 (70.8)
Chinese	4 (28.6)	2 (20.0)		5 (25.0)
Indian	1 (7.1)	0		1 (4.2)
Marital Status ^b^			0.393	
Single	3 (21.4)	4 (40.0)		7 (29.2)
Married	11(78.6)	6 (60.0)		17 (70.8)
Education Level ^b^				
Secondary	10 (71.4)	0	<0.001	10 (41.7)
Tertiary	4 (28.6)	10 (100.0)		14 (58.3)
Smoking Status ^b^			0.615	
Yes, currently smoke	2 (14.3)	3 (30.0)		5 (20.8)
Periodontitis Status ^b^			0.091	
Duration of diagnosis (year) ^c^	3.5 (4.3)	2.0 (1.0)		2.5 (2.0)

Data are presented as mean (SD) ^a^, frequency (%) ^b^, or median (IQR) ^c^.

**Table 2:** Changes in diabetes knowledge and lifestyle practices among the participants (*n* = 14).

Parameter	IG (*n* = 14)		CG (*n* = 10)		*p*-Value between Group ^a^ (IG-CG at W0)	*p*-Value within Group IG ^b^ (W0–W12)	*p*-Value within Group CG ^b^ (W0–W12)
	W0	W12	W0	W12			
Diabetes knowledge (%)	44.3 (12.7)	56.2 (11.6)	45.3 (14.7)	55.3 (10.9)	0.854	0.008	0.062
Body weight (kg)	70.2 (15.1)	68.8 (14.7)	69.5 (20.4)	69.7 (20.8)	0.916	0.039	0.825
Height (m)	1.65 (0.09)	-	1.62 (0.08)	-	0.407	-	-
Body mass index (kg/m^2^)	25.8 (5.1)	25.3 (4.9)	26.4 (7.6)	26.5 (7.7)	0.820	0.029	0.895
Waist circumference (cm)	90.9 (17.5)	90.6 (20.6)	88.4 (17.9)	88.7 (19.2)	0.736	0.929	0.747
Physical activity (MET-min/week)	1797.2 (1196.2)	1318.3 (591.0)	1629.7 (2394.5)	664.9 (422.8)	0.723	0.306	0.314
Oral health impact on daily performance (OIDP) *	6.5 (18.8)	0.0 (16.0)	2.0 (70.5)	0.0 (6.0)	0.928 ^c^	0.066 ^d^	0.075 ^d^
Energy (kcal/day)	2095 (411)	1490 (379)	2127 (560)	2032 (485)	0.875	<0.001	0.289
Carbohydrate (g/day)	276.7 (57.4)	181.7 (35.7)	292.6 (101.8)	227.9 (69.0)	0.629	<0.001	0.015
Protein (g/day)	70.0 (17.3)	62.0 (21.4)	70.8 (19.2)	77.0 (14.2)	0.909	0.202	0.190
Fat (g/day)	82.1 (26.7)	57.2 (26.2)	79.3 (25.2)	92.2 (27.1)	0.801	0.021	0.172
Added sugar intake (g/day) *	32.4 (24.9)	11.0 (21.6)	51.2 (57.7)	26.0 (89.6)	0.259	0.016 ^c^	0.646

Data are presented as mean (SD) or * median (IQR). ^a^ Independent *t*-test; ^b^ paired *t*-test; ^c^ Mann–Whitney; ^d^ Wilcoxon signed rank test.

**Table 3:** Overall feedback on the online diabetes wellness programme.

Theme	Sub-Theme	Quotes
Convenience	Save time and cost	*“It saves time and is cost-efficient. It didn’t bother me to drive and park my car” (S3, 45 years old).* *“I don’t need to leave home and go to the clinic at a specific time” (S9, 67 years old).*
	Feel more relaxed, comfortable, and safer	*“I feel so comfortable doing it while at home. Feel more relaxed during counselling and safer due to COVID-19” (S5, 63 years old).*
	Flexible	*“It has flexible schedule arrangement plus I don’t need to worry about traffic” (S9, 67 years old).* *“It is easy to attend and free to do at any time and place” (S7, 64 years old).*
Practical	Easy to follow	*“It is not too strict and easy to follow” (S7, 64 years old).* *“It is easy to remember the recommended changes because it also includes educational videos on the website” (S6, 61 years old).*
	Two-way learning	*“It is still face-to-face and allows me to learn more from consultants rather than self-learning through education videos” (S2, 52 years old).* *“A very good programme. I get a chance to ask specifically about dietary practice and oral health even in a pandemic” (S9, 67 years old).*
	Informative	*“The content was very informative. I have become more health-conscious than before” (S5, 63 years old).*
Preferred approach during pandemic		*“Good and new experience to use Google Meet for* *online counselling. It is very suitable to use now due to the pandemic outbreak” (S10, 62 years old).* *“I feel that it should continue as we are currently facing a pandemic and need to stay at home. It can be applied to any disease condition if they have good internet coverage” (S12, 51 years old).*
Increase awareness		*“It increases my awareness to regularly take my medicine and insulin, which I previously skipped as I did not know of the consequences. It guides me to slowly reduce and control my alcohol intake, which previously I was unaware that it could affect my blood sugar control and health” (S12, 51 years old).* *“I became more aware of the relationship between these two diseases (diabetes and periodontitis) that I realised it is important to manage both conditions simultaneously” (S2, 52 years old).*
Increase in knowledge		*“It helps me to increase my knowledge about my health, and I can feel my body healthier” (S6, 61 years old).*
Positive changes	Eating habits and exercise	*“The modules taught me how to eat correctly and also suggested suitable exercise specific duration. Now, it has already become my usual routine to exercise, and I learn to eat more moderately” (S12, 51 years old).*
	Weight	*“I’m very delighted as I have reduced my weight after joining this programme. The counselling helps to increase my determination to continue practicing a healthy lifestyle” (S6, 61 years old).*

**Table 4:** Barriers to implementing the recommended lifestyle changes.

Theme	Sub-Theme	Quotes
Internal	Lack of self- discipline	*“I find it hard to follow all recommended dietary intake due to my old eating habit. Even if I’m given more time, it depends on my self-discipline to practice it consistently every day” (S1, 59 years old).* *“I personally feel that the suggestions are not too strict and quite okay to follow, but it becomes too hard to accommodate with my usual lifestyle such as taking a less sweet drink or limit it to once daily” (S3, 45 years old).*
	Short duration	*“Hard to initiate changes and I need more time to practice it gradually. The 3-month duration was inadequate to practice all suggested changes” (S10, 62 years old).* *“I need more time (more than 3 months) to make all the suggested dietary changes as suggested during the counselling” (S4, 22 years old).* *“It is very easy to follow the suggestions, but I need more time to discipline myself” (S9, 67 years old).*
External	Lack of support	*“I find it hard to follow the suggestion because the people around me are unsupportive” (S3, 45 years old).*

## Data Availability

Not applicable.
